# The Bacterial Community Structure and Microbial Activity in a Traditional Organic Milpa Farming System Under Different Soil Moisture Conditions

**DOI:** 10.3389/fmicb.2018.02737

**Published:** 2018-11-14

**Authors:** Iván P. Moreno-Espíndola, María J. Ferrara-Guerrero, Marco L. Luna-Guido, Daniel A. Ramírez-Villanueva, Arit S. De León-Lorenzana, Selene Gómez-Acata, Elizabeth González-Terreros, Blanca Ramírez-Barajas, Yendi E. Navarro-Noya, Luis M. Sánchez-Rodríguez, Mariela Fuentes-Ponce, Juan U. Macedas-Jímenez, Luc Dendooven

**Affiliations:** ^1^Departamento de Producción Agrícola y Animal, Universidad Autónoma Metropolitana-Xochimilco, Mexico City, Mexico; ^2^Departamento de El Hombre y su Ambiente, Universidad Autónoma Metropolitana-Xochimilco, Mexico City, Mexico; ^3^Laboratory of Soil Ecology, ABACUS, Centro de Investigación y de Estudios Avanzados, Mexico City, Mexico; ^4^Cátedras Conacyt – Universidad Autónoma de Tlaxcala, Tlaxcala, Mexico

**Keywords:** C and N mineralization, crop rotation and maize monoculture, inorganic and organic fertilizer, milpa agricultural system, enzyme activity

## Abstract

Agricultural practices affect the bacterial community structure, but how they determine the response of the bacterial community to drought, is still largely unknown. Conventional cultivated soil, i.e., inorganic fertilization, tillage, crop residue removal and maize (*Zea mays* L.) monoculture, and traditional organic farmed soil “milpa,” i.e., minimum tillage, rotation of maize, pumpkin (*Cucurbita* sp.) and beans (*Phaseolus vulgaris* L.) and organic fertilization were sampled. Both soils from the central highlands of Mexico were characterized and incubated aerobically at 5% field capacity (5%FC) and 100% field capacity (FC) for 45 days, while the C and N mineralization, enzyme activity and the bacterial community structure were monitored. After applying the different agricultural practices 3 years, the organic C content was 1.8-times larger in the milpa than in the conventional cultivated soil, the microbial biomass C 1.3-times, and C and N mineralization 2.0-times (mean for soil incubated at 5%FC and FC). The dehydrogenase, activity was significantly higher in the conventional cultivated soil than in the milpa soil when incubated at 5%FC, but not when incubated at FC. The relative abundance of Gemmatimonadetes was larger in the conventional cultivated soil than in the milpa soil in soil both at 5%FC and FC, while that of Bacteroidetes showed an opposite trend. The relative abundance of other groups, such as Nitrospirae and Proteobacteria, was affected by cultivation technique, but controlled by soil water content. The relative abundance of other groups, e.g., FBP, Gemmatimonadetes and Proteobacteria, was affected by water content, but the effect depended on agricultural practice. For soil incubated at FC, the xenobiotics biodegradation and metabolism related functions were higher in the milpa soil than in the conventional cultivated soil, and carbohydrate metabolism showed an opposite trend. It was found that agricultural practices and soil water content had a strong effect on soil characteristics, C and N mineralization, enzyme activity, and the bacterial community structure and its functionality. Decreases or increases in the relative abundance of bacterial groups when the soil water content decreased, i.e., from FC to 5%FC, was defined often by the cultivation technique, and the larger organic matter content in the milpa soil did not prevent large changes in the bacterial community structure when the soil was dried.

## Introduction

Agriculture has a profound effect on soil ecosystem. Intensive traditional agriculture cultivation techniques, such as crop residue removal, monoculture, tillage and inorganic fertilizer application, reduce soil fertility and alter the soil microbial community structure (Mäder et al., 2002). Tillage breaks up aggregates, compacts soil and accelerates organic matter decomposition (Ussiri and Lal, 2009). Crop residue removal reduces soil organic matter content (Fuentes et al., 2009) and extensive inorganic fertilizer applications increases salt content and electrolytic conductivity (EC) (Rezapour, 2014). Soil microorganisms are highly sensitive to changes in soil characteristics due to these agricultural practices (Acosta-Martínez et al., 2008; Schipanski and Drinkwater, 2012; Vasseur et al., 2013). For instance, Navarro-Noya et al. (2013) showed that crop residue management affected the bacterial community and Carbonetto et al. (2014) found that both tillage and crop residue management altered the microbial community structure.

The sharp drop in soil fertility as a result of conventional agricultural techniques has promoted agricultural practices that halt this decrease or even reverse it (Mäder et al., 2002). Minimum tillage, crop residue retention and crop rotation have been proposed to stop the decline in soil fertility (Govaerts et al., 2006). The Milpa system, a traditional organic farming agrosystem practiced in Mesoamerica, has been promoted in Mexico to reverse the decline in soil fertility. Milpa combines rotation of maize (*Zea mays* L.), pumpkin (*Cucurbita sp.*) and beans (*Phaseolus vulgaris* L.) with organic material input and soil conservation practices (Zimmerer, 2010). As a consequence, plant growth is better and yields are higher in the milpa system than in the conventional system. For instance, the biomass production in the Milpa system was 10,224 kg ha^-1^ and only 4,872 kg ha^-1^ in the conventional system in 2012, while in 2013 it was 10,505 kg ha^-1^ in the milpa system and 8,834 kg ha^-1^ in the conventional one.

The retention of crop residue and/or the application of organic material prevents wind and water erosion, improves soil structure and maintains the soil water longer. Water availability regulates microbiological activity and determines soil microbial communities (Falkowski et al., 2008). Microorganisms play a key role in the soil where they are responsible for most of the biogeochemical cycles, mineralization of organic matter and the release of plant nutrients. On the one hand, when the soil water content decreases the bacterial community structure changes and microbial activity is reduced (Santos-Medellín et al., 2017). For instance, Sardans and Peñuelas (2005) found a decrease in urease and protease activities when soil water content decreased. On the other hand, when the soil moisture content is high, O_2_ diffusion is inhibited and microbial activity is reduced. Anaerobic sites are formed in soil favoring facultative anaerobes or anaerobes altering the soil microbial composition (Skopp et al., 1990; Moldrup et al., 2001; Schimel et al., 2007).

It was hypothesized that the higher organic matter content in the Milpa soil would protect microorganisms better against drying compared to a conventional cultivated soil. Therefore, soil was taken from an organic milpa system and soil under conventional practice. The soils were adjusted to field capacity (FC) and 5% of FC (5%FC) and the effect of the agricultural practices and soil water content on the bacterial community structure was monitored in an aerobic incubation of 45 days. The study of soil microbiology is essential to design sustainable agricultural production systems that are alternatives to conventional practices, which together with industrial activities have altered biogeochemical cycles, particularly the carbon cycle (Intergovernmental Panel on Climate Change [IPCC], 2014).

## Materials and Methods

### Soil Sampling and Experimental Site

The experimental site is located in the municipality of Cocotitlán in the east of the State of Mexico, Mexico, 19°12′18” and 19°14′33” north latitude; and 98°49′46” and 98°52′52” west longitude at an altitude of 2,300 m.a.s.l. The temperate sub-humid climate is of the C(w1)(w) type (García, 2004). The rainy season runs from May to October with an annual rainfall of 784 mm. The soil is Vitric eutric epiarenic (WRB classification). The study site is included in the Sierra Nevada Research Program (PISN) of the “*Universidad Autónoma Metropolitana* (Mexico).”

Samples were collected from soil under two different agricultural practices. First, soil was sampled from a conventional system, i.e., normal tillage, crop residue removal, inorganic fertilizer application, herbicide application and maize monoculture for 3 years. In the conventional treatment, machinery was used for soil preparation (deep fallow (30–35 cm) and harrow (15 cm), as well as for furrowing and sowing. Second, soil was sampled from an organic milpa system, i.e., zero tillage, 100% retention of crop residues, organic fertilizer application, weed management and rotation of maize, pumpkin and beans for 3 years. No tillage was applied in the milpa system, all of the maize residue from the previous crop were retained and weed control was performed manually. Seed sowing was done using a hand tool known as “coa.” In 2012, the organic fertilizer application in the milpa system was composted cow dung added to each plant (4.4 t ha^-1^). In 2013 and 2014, the first organic fertilizer applied contained a consortium of microorganisms and 500 kg ha^-1^ basalt rock dust, while the second organic fertilizer application consisted of composted sheep dung (3.3 t ha^-1^) and chicken manure (1.1 t ha^-1^). Oak forest mulch was used to produce the microbial consortium. This consortium included phototrophic and lactic acid producing bacteria, as well as Actinomycetes, yeasts and fermentation fungi (Higa, 2013). A mix of 100 kg of forest mulch, 100 kg rice bran and 5 L molasses was placed in a 200 L container and left to mature for 28 days. Approximately 200 kg ha^-1^ of this mixture was applied to the milpa soil. The conventional cultivated soil was fertilized with urea (243.5 kg ha^-1^) and calcium triple superphosphate (50 kg ha^-1^), while a herbicide [Hierbamina (2,4-D)] was applied at a rate of 12.5 l y^-1^ ha^-1^. Both inorganic and organic fertilizations are applied in the middle of July. For instance in 2013, inorganic and organic fertilizations were applied on the 18th of July.

Five sub-samples of soil were spade sampled from the 0–10 cm layer of three plots from both cultivation systems on 27th January 2014 (Supplementary Figure S1). The five soil samples from each plot were pooled so that six soil samples of 2 kg were obtained, i.e., from three plots and two agricultural practices. The soil was collected in sterile bags and kept on ice while transported to the laboratory. This field based replication was maintained in the laboratory experiment to avoid pseudoreplication (Hurlbert, 1984).

### Aerobic Incubation

Twelve sub-samples of 20 g soil from each plot (*n* = 3) and cultivation systems (*n* = 2) were added separately to 120 ml glass flasks. Two different treatments were applied to each soil of each cultivation system. Six sub-samples of both cultivation systems and three plots were adjusted to 5% field capacity (5%FC) by applying 3.9 ml distilled H_2_O to the milpa soil and 5 ml to the conventional tilled soil. The other six soil samples were adjusted to FC by applying 78 ml distilled water to the milpa soil and 101 ml to the conventional tilled soil so that a similar % of water content (5%FC or FC) was obtained in both soils. Each flask was placed in a 1-l glass jar containing a 25 ml flask with 20 ml 0.5 M NaOH to capture the evolved CO_2_ and a 25 ml flask filled with distilled water to avoid desiccation of the soil during incubation, and closed air-tight. After 0, 1, 3, 7, 14, and 45 days, the jars were opened, the 25 ml flask with 0.5 M NaOH taken out and analyzed for the trapped CO_2_. The soil was removed from the flask, 6 g was extracted for DNA and 24 g was used to determine enzymatic activity as described below, while the rest was extracted for mineral N (NH_4_^+^, NO_2_^-^ and NO_3_^-^) with 100 ml 0.5 M K_2_SO_4_. The K_2_SO_4_ extract was then analyzed for mineral N on a San Plus System-SKALAR automatic analyzer (Skalar, Breda, Netherlands) (Mulvaney, 1996).

### Soil Characterization and Enzyme Activity

Soil pH was measured in 1:2.5 soil-H_2_O suspension using a glass electrode (Thomas, 1996). The NH_4_^+^, NO_2_^-^ and NO_3_^-^ in the K_2_SO_4_ extracts were determined colorimetrically on a San Plus System–SKALAR automatic analyzer (Mulvaney, 1996). The CO_2_ trapped in 1 M NaOH was determined by titration with 0.1 M HCl (Jenkinson and Powlson, 1976). The soil microbial biomass carbon was determined as described by Vance et al. (1987).

The potential activity of dehydrogenase, acid phosphatase, urease and protease (PRO), enzymes involved in the degradation of organic material was determined with spectrophotometric methods. Dehydrogenase and protease activity was measured as described by Rangaswamy et al. (1994), acid phosphatase determined based on the method of Tabatabai (1994) and urease activity with the technique reported by Kandeler et al. (1999b). Corresponding calibration curves were generated for each enzymatic activity and each test included a control and six replicates.

### DNA Extraction and PCR Amplification of Bacterial 16S rRNA Genes

Twelve sub-samples of 0.5 g soil were washed with pyrophosphate 0.15 M and treated with a phosphate buffer pH 8. The DNA was extracted from the 12 washed soil samples using three different techniques, i.e., four sub-samples of 0.5 g were used for each technique. The first technique used was based on the method reported by Valenzuela-Encinas et al. (2008), the second by Griffiths et al. (2000) and the third by Hoffman and Winston (1987). As such, 6 g soil (four subsamples of 0.5 g soil and three different techniques) was extracted for DNA from each plot (*n* = 3). Overall, 18 g soil was extracted for DNA from each treatment (*n* = 2) at each sampling day. The DNA extracts were purified with chloroform-isoamylic alcohol 24:1, precipitated with polyethylene glycol 8000 and washed with 70% ethanol. The DNA quality was determined on an agarose gel 0.8% and a NanoDrop (A_260_/A_280_ > 1.8).

The V1–V3 region of the 16S rRNA bacterial genes was amplified with 10-pb barcoded primers 8-F (5′-AGA GTT TGA TCI TGG CTC A-3′) and 556-R (5′-TGC CAG IAG CIG CGG TAA-3′) and containing the A and B 454 FLX adapters (Navarro-Noya et al., 2013). The PCR reactions, purification and quantification of the DNA were done as previously described by Navarro-Noya et al. (2013). Sequencing was done by Macrogen Inc. (DNA Sequencing Service, Seoul, Korea) using a Roche 454 GS-FLX Titanium System pyrosequencer (Roche, Mannheim, Germany). The QIIME version 1.9.0 software pipeline was used to analyze the pyrosequencing data (Caporaso et al., 2010) and details of the analysis of the pyrosequencing data can be found in Navarro-Noya et al. (2013).

### Metabolic Capacities of the Bacterial Communities

The metabolic activities of the bacterial communities were predicted using METAGENassist statistical tools for comparative metagenomic analysis based on the taxonomic assignations of the clustered operational taxonomic units (OTUs)^1^ (Arndt et al., 2012). Data filtering was based on interquartile range (IQR), row normalization by sum and column normalization based on autoscaling. The functional profiling of the bacterial communities was predicted by ancestral state reconstruction using PICRUSt 1.1.1 software (PICRUSt^2^) (Langille et al., 2013) and the Kyoto encyclopedia of genes and genomes (KEGG) (Kanehisa et al., 2016).

### Phylogenetic and Statistical Analysis

The taxonomic distribution estimates at different levels was done using the taxonomy assignation at a confidence threshold of 80% by the naïve Bayesian rRNA classifier from the Ribosomal Data Project^3^ (Wang et al., 2007). Diversity and species richness estimators were calculated from a 6,160 sequence rarified biom table to avoid bias due to the differences in the sampling effort. Details can be found in Navarro-Noya et al. (2013).

Cumulative production of CO_2_ was regressed on elapsed time using a linear regression model which was forced to pass through the origin but allowed different slopes (production rates) for each treatment. Significant differences between milpa and conventional cultivated soil incubated at 5%FC or FC for emission of CO_2_ were determined using PROC MIXED (SAS Institute, 1989). Significant differences between the mineral N, biomass C, enzyme activity and soil characteristics in soil as a result of water content, agriculture cultivation technique and their interaction were determined by analysis of variance (ANOVA) (parametric distributed) and based on the least significant difference using the general linear model procedure (PROC GLM, SAS Institute, 1989).

A non-parametric test was applied to determine if the effect of management practice (conventional versus milpa), water content (5%FC versus FC) and their interaction was significant using a two-way factorial design in R (R Development Core Team, 2008; Feys, 2016). The t2way test of the WRS2 package (A collection of robust statistical methods) was used (Mair and Wilcox, 2017). Abundance of the different bacterial taxonomic levels was explored separately with a principal component analysis (PCA) and constrained analysis of principal coordinates (CAP) was used to explore bacterial groups and soil characteristics done with the vegan package in R (Oksanen et al., 2017). Heatmaps were constructed with pheatmap package (Kolde, 2015).

### Data Accessibility

Raw sequences have been submitted as a sequence read archive (SRA) to the NCBI under BioProject accession number PRJNA317233 and the Biosamples SAMN04858132–SAMN04858203.

## Results

### Soil Characteristics

After 3 years of contrasting agricultural practices, the organic C content in the sandy loam soil had increased significantly 1.8-times in the milpa system compared to the conventional agricultural practices and total N 1.5-times (*p* < 0.05) (Table 1). None of the other measured soil characteristics, however, was different between the two agricultural practices (Table 2).

**Table 1:** Characteristics of soil cultivated conventionally, i.e., conventional tillage, crop residues removal, chemical fertilizer and herbicide application and monoculture of maize (*Zea mays* L.), or cultivated with an organic milpa system, i.e., zero tillage, retention of crop residues, organic fertilizer application, weed management and crop rotation of maize, pumpkin (*Cucurbita* sp.) and beans (*Phaseolus vulgaris* L.), for 3 years.

		EC ^a^	FC ^b^	WC ^c^	Total C	Total N	Clay	Sand	Loam	USDA textural
Treatment	pH	(dS m^-1^)				(g kg^-1^ soil)				classification
Conventional	6.1 A	0.37 A	480 A	57 A	6.1 B	0.57 B	60	530	410	Sandy loam
Milpa	6.2 A	0.33 A	512 A	69 A	10.7 A	0.83 A	60	530	410	Sandy loam
*F*-Value	0.35	0.00	0.25	0.96	20.50	32.00	ND ^c^	ND	ND	
*p*-Value	0.6158	0.9906	0.645	0.384	0.0106	0.0048	ND	ND	ND	

*^a^ EC, electrolytic conductivity; ^b^ FC, field capacity; ^c^ ND, not determined.*

**Table 2:** Characteristics of soil cultivated conventionally, i.e., conventional tillage, crop residues removal, chemical fertilizer and herbicide application and monoculture of maize (*Zea mays* L.), or cultivated according to an organic milpa system, i.e., zero tillage, retention of crop residues, organic fertilizer application, weed management and crop rotation of maize, pumpkin (*Cucurbita* sp.) and beans (*Phaseolus vulgaris* L.), for 3 years.

	CEC ^a^	Olsen P	Potassium	Calcium	Magnesium		Sodium	Aluminum
Treatment	(cmoles+ kg^-1^)	(mg kg^-1^ soil)						
Conventional	12.6 A ^b^	7.7 A	129 A	1038 A		200 A	27	ND ^c^
Milpa	24.9 A	16.7 A	226 A	1048 A		218 A	24	ND
MSD	14.3	13.7	615	200		69	32	
*F*-Value	5.71	3.07	0.88	2.06		3.94	0.10	
*p*-Value	0.0752	0.1545	0.4471	0.2877		0.1857	0.7806	

*^a^ CEC, cation exchange capacity; ^b^ values with the same capital letter are not significant different between the soils, i.e., within the columns; ^c^ ND, not detectable.*

### C and N Mineralization and Microbial Biomass C

**FIGURE 1:**
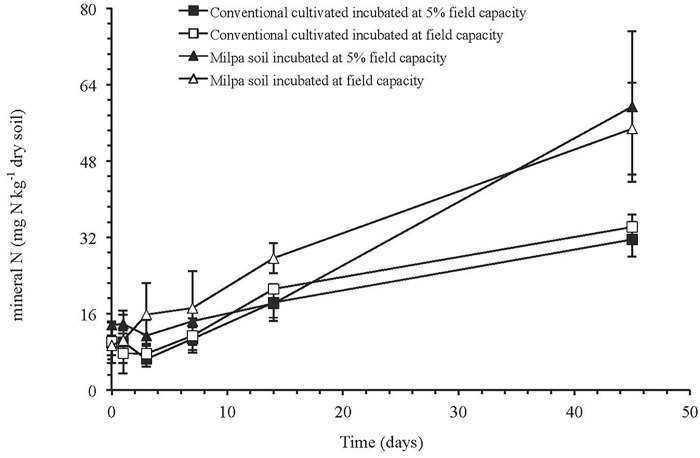
Concentrations of mineral N (mg N kg^-1^ dry soil) (sum of NH_4_^+^, NO_2_^-^ and NO_3_^-^) in milpa soil or soil cultivated conventionally incubated at 5% of field capacity or field capacity for 45 days.

The CO_2_ emission rate was in the order: milpa soil incubated at FC > milpa soil incubated at 5%FC > conventional cultivated soil at FC = conventional cultivated soil at 5% FC (*p* < 0.05) (Table 3). The amount of C mineralized after 45 days was 1.9-times larger for the milpa soil incubated at 5%FC than in the conventional cultivated soil at 5%FC and it was 2.1-times larger when the soils were incubated at FC. The effect of agricultural practice on the CO_2_ emitted after 45 days was significant (*p* < 0.05), but water content and its interaction with agricultural practice had no significant effect (Supplementary Table S1). The soil microbial biomass C was significantly lower in conventional cultivated soil incubated at 5%FC than the milpa soil incubated at 5%FC or FC (*p* < 0.05). Agricultural practice had a highly significant effect on the soil microbial biomass C (*p* < 0.001), but water content and its interaction with agricultural practice not (Supplementary Table S1). The amount of N mineralized was similar when the soils were incubated at 5%FC or FC, but it was twice as high in the milpa soil (45.6 mg kg^-1^ dry soil) than in the conventional cultivated soil (22.8 mg kg^-1^ dry soil) (Figure 1).

**Table 3:** The CO_2_ production rate and microbial biomass C in soil cultivated conventionally, i.e., conventional tillage, crop residues removal, chemical fertilizer and herbicide application and monoculture of maize (*Zea mays* L.), or an organic milpa system, i.e., zero tillage, retention of crop residues, organic fertilizer application, weed management and crop rotation of maize, pumpkin (*Cucurbita* sp.), and beans (*Phaseolus vulgaris* L.), for 3 years.

		CO_2_ emitted		Biomass C
Treatment	Water content	(mg C kg^-1^ soil day^-1^)		(mg C kg^-1^)
Conventional	5%FC ^a^	6.41 C ^b^		255 B
	FC	7.14 C		350 AB
Milpa	5%FC	11.75 B		399 A
	FC	15.11 A		396 A
	SEE ^c^	0.79	MSD ^d^	113
	*F*-Value	53.86	*F*-Value	4.88
	*p*-Value	<0.0001	*p*-Value	0.0039

*^a^ FC, field capacity; ^b^ values with the same capital letter are not significantly between the treatments, i.e., within the column (p < 0.05); ^c^ SEE, standard error of the estimates; ^d^ MSD, minimum significant difference at p < 0.05.*

### Potential Enzyme Activities

The enzyme activity was generally higher in soil incubated at 5%FC than incubated at FC (Table 4). In both treatments, dehydrogenase and urease activity was higher significantly in soil incubated at 5%FC than at FC (*p* < 0.05). Dehydrogenase activity was higher significantly in conventional cultivated than in the milpa soil incubated at 5%FC (*p* < 0.05) and protease activity in the conventional cultivated soil at 5%FC compared to the milpa soil incubated at FC. The urease activity was higher in the conventional cultivated and milpa soil incubated at 5%FC than at FC. The acid phosphatase activity was significantly higher in conventional cultivated soil incubated at 5%FC than at FC (*p* < 0.05).

**Table 4:** Enzymatic activity in soil cultivated conventionally, i.e., conventional tillage, crop residues removal, chemical fertilizer and herbicide application and monoculture of maize (*Zea mays* L.), or cultivated with the organic milpa system, i.e., zero tillage, retention of crop residues, organic fertilizer application, weed management and crop rotation of maize, pumpkin (*Cucurbita* sp.) and beans (*Phaseolus vulgaris* L.), for 3 years.

	Water content	Dehydrogenase	Urease	Protease	Acid phosphatase
Treatment	(% field capacity)	(μg INTF g^-1^ h^-1^)	(μmol N-NH_4_^+^ g^-1^ h^-1^)	(μmol tyrosine g^-1^h^-1^)	(μmol *p*-nitrophenol g^-1^ h^-1^)
Conventional	5	15.9 A ^a^	3.12 A	1.57 A	1.30 A
	100	5.9 C	1.01 B	0.87 AB	1.04 B
Milpa	5	8.8 B	2.99 A	1.38 AB	1.21 AB
	100	2.8 C	1.25 B	0.69 B	1.19 AB
MSD		2.9	1.07	0.74	0.24
*F*-Value		50.31	15.05	4.41	2.94
*p*-Value		<0.0001	<0.0001	0.0068	0.0394

*^a^ Values with the same capital letter are not significantly between the treatments, i.e., within the column (p < 0.05), ^b^ MSD, minimum significant difference at p < 0.05.*

Water content had a significant effect on the urease, protease, and acid phosphatase activity C (*p* < 0.05), but agricultural practice and its interaction with water content not (Supplementary Table S1). Water content, agricultural practice and its interaction with water content had a significant effect on dehydrogenase activity.

### Bacterial Community Structure

Overall, 96,485 good quality sequences were extracted from the milpa and conventional cultivated soil with a total of 9,167 OTUs. The rarification curves of the number of sequences versus the number of OTUs for each of the two soils incubated at 5%FC or FC was asymptotic indicating that an increase in the number of sequences extracted would only marginally increase the number of OTUs obtained (Supplementary Figure S2). The number of observed species, Chao1 estimator, and the Shannon and Simpson diversity indexes were similar for the conventional cultivated and the milpa soil incubated at 5%FC and FC (Table 5). The number of OTUs, Chao1 estimator and the Shannon diversity, however, tended to decrease with incubation time (Supplementary Table S2).

**Table 5:** Non-parametric analysis of the effect of soil cultivation technique and soil moisture content and their interaction on the alpha diversity of the bacterial populations in soil cultivated conventionally, i.e., conventional tillage, crop residues removal, chemical fertilizer and herbicide application and monoculture of maize (*Zea mays* L.), or an organic milpa system, i.e., zero tillage, retention of crop residues, organic fertilizer application, weed management and crop rotation of maize, pumpkin (*Cucurbita* sp.) and beans (*Phaseolus vulgaris* L.), for 3 years.

Treatment	Water content	Observed species	Chao1 richness estimator	Simpson diversity index	Shannon diversity index
Conventional	5%FC ^a^	1662 ^b^	3041	0.9974	9.683
	FC ^c^	1340	2308	0.9930	8.993
Milpa	5%FC	1390	2481	0.9965	9.312
	FC	1539	2660	0.9964	9.432
Treatment (Tre) (*p*-value) ^d^		0.592	0.364	0.533	0.855
Water content (WC) (*p*-value)		0.224	0.032	0.278	0.163
Interaction Tre × WC (*p*-value)		0.007	0.002	0.296	0.064

*^a^ 5%FC, soil incubated at 5% of field capacity; ^b^ values obtained after 0, 1, 3, 7, 14, and 45 days (see Supplementary Table S2) were averaged per plot (n = 3); ^c^ FC, soil incubated at field capacity; ^d^ non-parametric t2way analysis [Package: WRS2, R Package (Mair and Wilcox, 2017)]. Soil was incubated aerobically at 5% of field capacity of field capacity for 45 days.*

Phylotypes belonging to 35 different bacterial phyla, 125 classes, 241 orders, and 405 families in the milpa and conventional tilled soil. Variations in the relative abundance of the bacterial groups, e.g., Phyla, were small generally during the aerobic incubation (Supplementary Figure S3).

**FIGURE 2:**
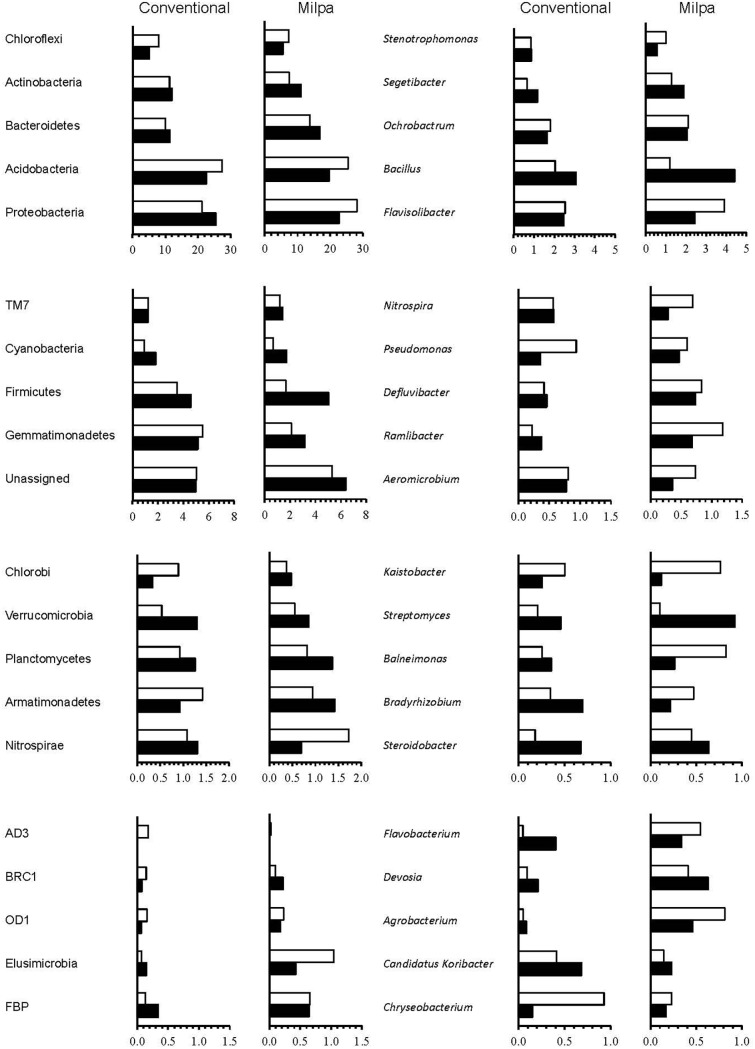
Relative abundance of the most abundant bacterial phyla and genera in the milpa and the conventional cultivated soil incubated at field capacity (FC) (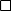) and 5%FC (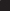).

The agricultural systems applied affected numerous bacterial groups, as did the soil water content (Figure 2 and Supplementary Table S3). The effect that the agricultural practices had on the relative abundance of the bacterial groups was controlled mostly by the soil water content, but not always. On the one hand, the relative abundance of the Gemmatimonadetes was larger in the conventional cultivated soil than in the milpa soil independent of water content, while that of the Bacteroidetes showed an opposite trend (*p* < 0.05). On the other hand, the relative abundance of the Bacteroidetes, FBP and Proteobacteria was larger in the milpa soil than in the conventional soil when incubated at FC, but not when incubated at 5%FC (*p* < 0.05). The relative abundance of the Nitrospirae was lower in the Milpa soil than in the conventional soil when incubated at 5%FC, but not when incubated at FC (*p* < 0.05). The latter was found at all bacterial levels and occurred often. For instance, of the 20 most abundant bacterial genera, the relative abundance of *Candidatus* Koribacter was significantly higher in the conventional soil compared to the milpa soil independent of the soil water content while that of *Bradyrhizobium* only when the soil was incubated at 5%FC (Figure 2 and Supplementary Table S3). The relative abundance of *Agrobacterium*, *Flavobacterium* and *Ramlibacter* and *Segetibacter* was larger in the milpa soil compared to the conventional soil when incubated at FC, but not when incubated at 5%FC.

The effect soil water content had on the relative abundance of the bacterial group was controlled often by the agricultural practices applied to the soil, but not always (Figure 2 and Supplementary Table S3). On the one hand, the relative abundance of the Acidobacteria was lower in the milpa and conventional soil incubated at 5%FC than at FC (*p* < 0.05). On the other hand, the relative abundance of the Actinobacteria, Cyanobacteria, Firmicutes, and Gemmatimonadetes was larger in the milpa soil incubated at 5%FC than when incubated at FC and that of the FBP and Proteobacteria in the conventional soil, while the relative abundance of the Chloroflexi was larger in the conventional soil incubated at FC than at 5%FC (*p* < 0.05). This phenomenon was found at all bacterial levels and occurred often. For instance, of the 20 most abundant bacterial genera, the relative abundance of *Bradyrhizobium* was larger in the conventional soil incubated at 5%FC than when incubated at FC and that of *Bacillus* in the milpa soil (*p* < 0.05) (Figure 2 and Supplementary Table S3). The relative abundance of *Chryseobacterium*, *Flavisolibacter*, and *Kaistobacter* was larger in the milpa soil incubated at 5%FC than when incubated at FC (*p* < 0.05).

**FIGURE 3:**
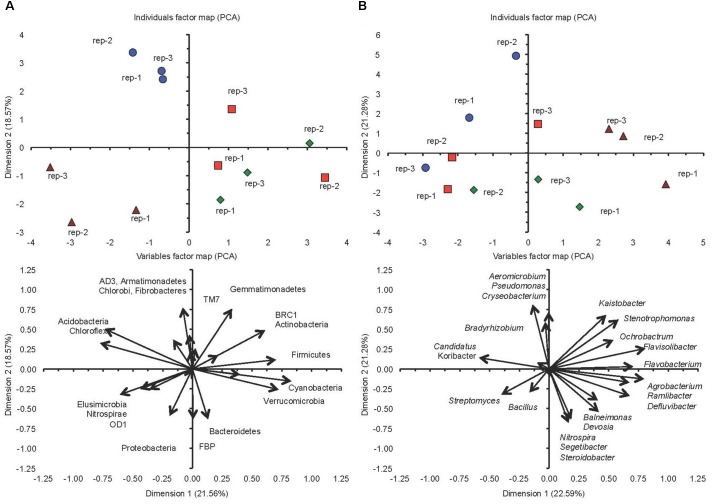
**(A)** Principal component analysis with the different bacterial phyla and **(B)** the 20 most abundant genera in soil cultivated conventionally incubated at 5% of field capacity (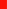) or field capacity (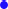) or milpa soil incubated at 5% of field capacity (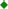) or field capacity (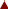) incubated aerobically for 45 days.

The PCA separated the two soils incubated at FC clearly, but did not separate them when incubated at 5%FC considering the different phyla (Figure 3A). The conventional cultivated soil when incubated at FC was characterized by a negative PC1 and a positive PC2, i.e., a larger relative abundance for the AD3, Armatimonadetes, Chlorobi, and Fibrobacteres, while the milpa soil incubated at FC was characterized by a more negative PC1 and a negative PC2, i.e., a larger relative abundance for the Elusimicrobia, Nitrospirae and OD1. The conventional cultivated soil and the milpa soil incubated at 5%FC were characterized by a positive PC1, i.e., a larger relative abundance for the Cyanobacteria, Firmicutes, and Verrucomicrobia. Considering the different bacterial genera in the PCA, then the separation of the different treatments was more outspoken and even the milpa and conventional cultivated soil incubated at 5%FC were separated (Figure 3B). The milpa soil incubated at FC had a higher relative abundance of genera such as the *Agrobacterium*, *Flavisolibacter*, *Flavobacterium*, and *Ramlibacter* than the conventional soil incubated at FC. The conventional soil incubated at FC was characterized by a larger relative abundance of genera such as *Aeromicrobium*, *Candidatus* Koribacter, *Chryseobacterium*, and *Pseudomonas*. The bacterial community structure considering the 20 abundant genera were for the milpa and conventional soil incubated at 5%FC were between the soils incubated at FC. The effect of soil water content on the bacterial community structure was more accentuated in the milpa soil than in the conventional cultivated soil.

**FIGURE 4:**
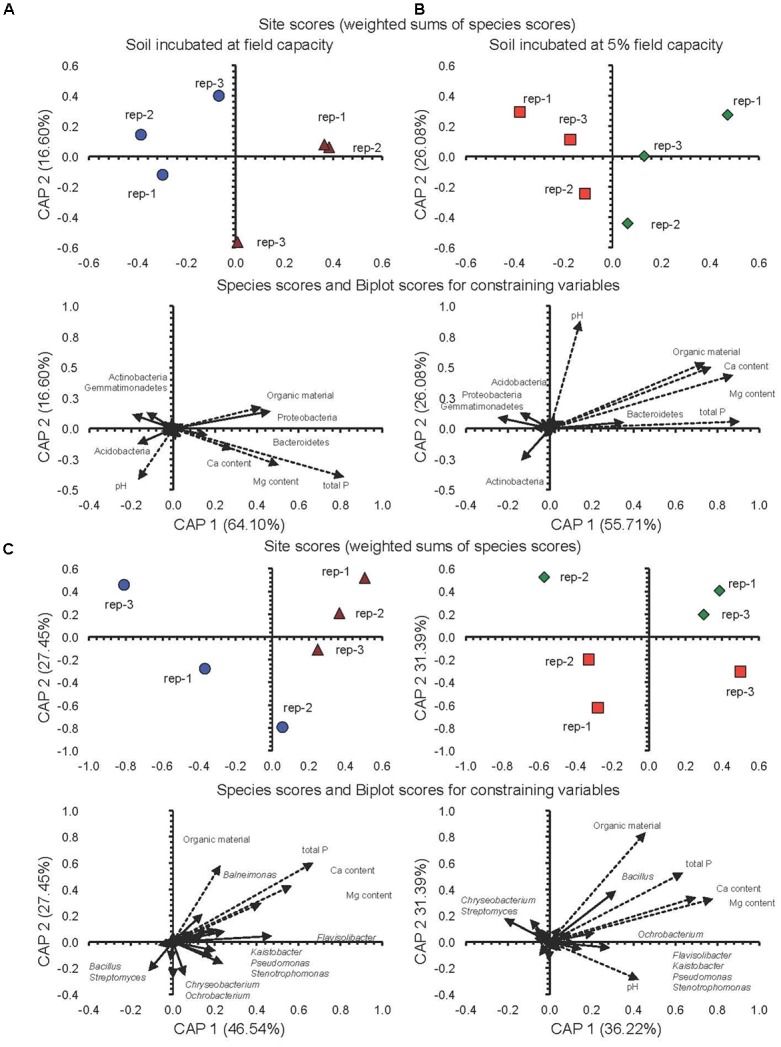
**(A)** Canonical analysis of principal coordinates (CAP) with the different bacterial phyla and characteristics of conventional cultivated soil incubated at field capacity (FC) (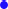) or milpa soil incubated at FC (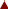), **(B)** conventional cultivated soil (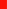) or the milpa soil (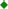) incubated aerobically at 5%FC for 45 days, **(C)** CAP with the different bacterial genera and characteristics of conventional cultivated soil incubated at field capacity (FC) (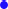) or milpa soil incubated at FC (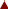), **(D)** conventional cultivated soil (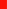) or the milpa soil (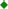) incubated aerobically at 5%FC for 45 days.

The CAP analysis considering soil characteristics and the relative abundance of the 20 most important phyla and genera of the soils incubated at FC or 5%FC separated the conventional system from the milpa system (Figures 4A–D). For instance, the relative abundance of the Bacteroidetes and Proteobacteria was higher in the milpa soil with a higher organic matter, P, Ca, and Mg content incubated at FC than in the conventional tilled soil while that of the Acidobacteria, Actinobacteria, and Gemmatimonadetes showed an opposite trend (Figure 4A). A similar pattern emerged for soil incubated at 5%FC except for the Proteobacteria as their relative abundance was higher in the conventional tilled soil than in the milpa soil (Figure 4B). The ANOSIM analysis confirmed that cultivation technique, water content at which the soils were incubated and incubation time all had a significant effect on the bacterial population structure (Supplementary Table S4).

### Bacterial Metabolic Capacity

**FIGURE 5:**
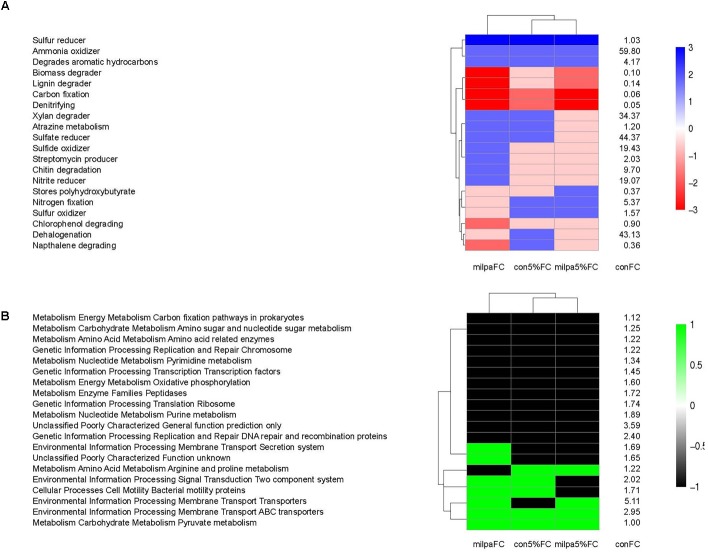
**(A)** The METAGENassist metabolic functions. **(B)** The KEGG orthologs metabolic functions in the conventional cultivated soil incubated at field capacity (conFC) and the heat map of the ratios comparing the metabolic functions in conFC with those in the conventional cultivated soil incubated at 5% field capacity (con5%FC), the milpa soil incubated at 5% field capacity (mil5%FC), and the milpa soil incubated at field capacity (milFC). Ratio –3: the metabolic function was ≥5 times lower than in conFC treatment, Ratio –2: the metabolic function was <5 times but ≥2 times lower than in conFC treatment, Ratio –1: the metabolic function was <2 times but >1 lower than in the conFC treatment, Ratio 0: the metabolic function was similar as in the conFC treatment, Ratio 1: the metabolic function was ≥1 times greater but <2 greater than in the conFC treatment, Ratio 2: the metabolic function was ≥2 times greater but <5 times greater than in conFC treatment, and Ratio 3: the metabolic function was ≥5 times greater than in conFC treatment.

The conventional cultivated soil incubated at 5%FC and FC resembled each other considering the METAGENassist metabolic functions analysis and the milpa soil incubated at 5%FC resembled them the least (Figure 5A). Differences in the METAGENassist metabolic functions between the soils were noticeable for atrazine metabolism, chitin degradation, chlorophenol and naphthalene degrading, nitrite and sulfur reducer, nitrogen fixation, and streptomycin producer. Changes in the KEGG orthologs function prediction due to agricultural practices and soil water content were small (Figure 5B). The KEGG orthologs metabolic functions were defined by soil water content, i.e., the milpa soil incubated at 5%FC resembled the conventional tilled soil incubated at 5%FC and the same occurred for soil incubated at FC.

**FIGURE 6:**
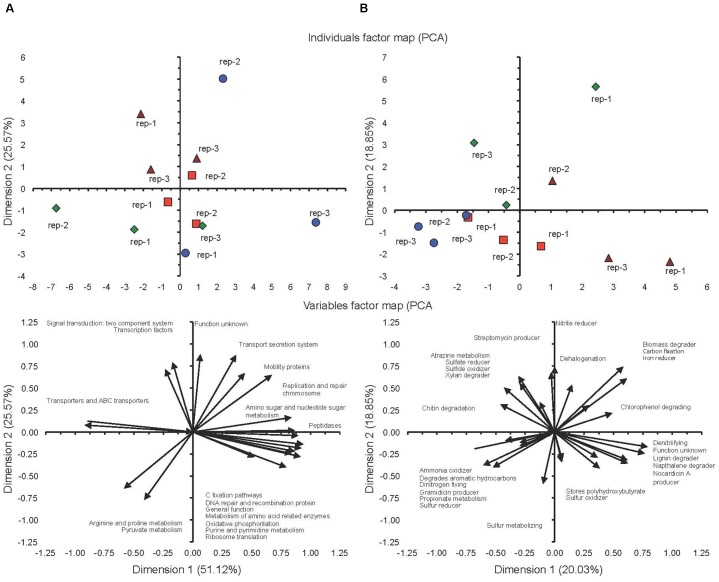
**(A)** Principal component analysis with the KEGG orthologs metabolic functions. **(B)** The METAGENassist metabolic functions in soil cultivated conventionally incubated at 5% of field capacity (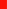) or field capacity (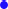) or milpa soil incubated at 5% of field capacity (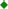) or field capacity (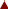) incubated aerobically for 45 days.

The PCA considering the KEGG orthologs function prediction showed a difference between the milpa soil and the conventional cultivated soil at both 5%FC and FC, although the latter was characterized by large variations between the plots when incubated at FC (Figure 6A). The milpa soil incubated at FC had more transporters and ABC transporters than the conventional cultivated soil incubated at FC and more arginine, proline and pyruvate metabolism when incubated at 5%FC. The effect of water content on the KEGG orthologs function prediction was more accentuated in the milpa soil than in the conventional cultivated soil. The PCA considering the METAGENassist metabolic functions showed a difference between the milpa soil and the conventional cultivated soil at both 5%FC and FC (Figure 6B). The PCA with the METAGENassist metabolic functions showed a clear effect of water content in both soils, but the effect was opposite.

### Ecological Coherence of High Bacterial Ranks

**FIGURE 7:**
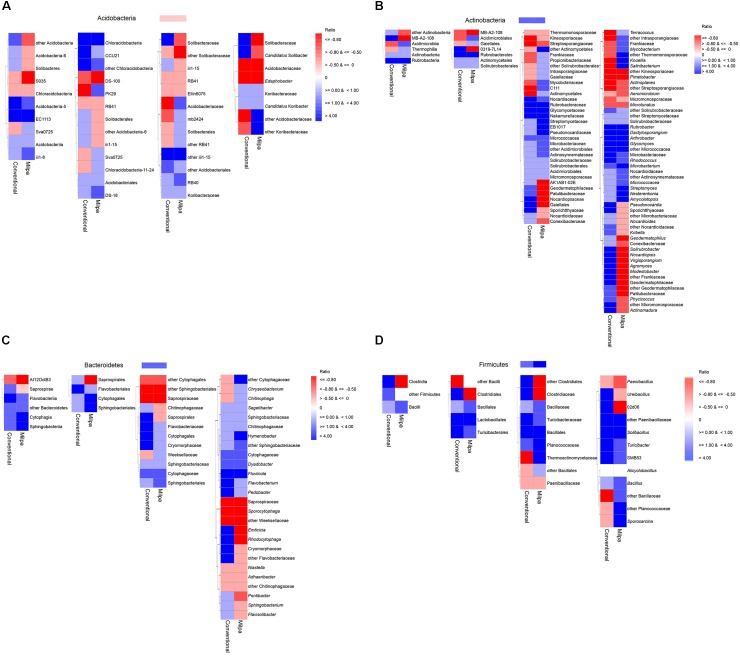
Heat map of the effect of water content on the relative abundance of different bacterial taxonomic groups of the **(A)** Acidobacteria, **(B)** Actinobacteria, **(C)** Bacteroidetes, and **(D)** Firmicutes in the conventional or milpa cultivated soil [Ratio = (the relative abundance of the bacterial group in soil at 5% field capacity (FC) minus the relative abundance of the bacterial group in the soil incubated at FC)/the relative abundance of the bacterial group in the soil incubated at FC].

Four bacterial phyla, i.e., Acidobacteria, Actinobacteria, Bacteroidetes, and Firmicutes, were selected to study their ecological coherence with changing soil water content (Figures 7A–D). The relative abundance of the phylum Acidobacteria decreased (<2 times) when the water content was 5%FC compared to that in both soils at FC. The response of the lower taxonomic levels to changes in the water content depended often on the bacterial group and/or soil characteristics. At the class level only three acidobacterial groups responded as on the phylum level in the conventional soil and 5 in the milpa soil. However, the relative abundance of only two acidobacterial classes, i.e., [Chloracidobacteria] and S035, decreased in both soils with a water content 5%FC compared to FC, while that of four increased. A similar pattern emerged when other bacterial groups, such as Actinobacteria, Bacteroidetes, or Firmicutes, were considered.

## Discussion

### Soil Characteristics

Although the two agricultural cultivation techniques applied at the experimental site are different in their concept, the effect on soil characteristics was small, except for organic C content, after only 3 years. As could be expected, the organic C content was different between the two treatments. Organic management practices are opposite, i.e., the conventional cultivation system includes tillage without crop residues retention while the milpa system promotes minimum tillage, retention of crop residues and organic fertilizer application. Tillage disrupts soil aggregates and renders organic material protected available to soil microorganism (Six et al., 2004). Removal of crop residues limits the replenishment of soil organic matter (Huggins et al., 2007). A combination of crop residue removal and tillage decreases soil organic matter with all its deteriorating effects, i.e., erosion, lack of soil structure, a reduction in available plant nutrients, increased evaporation, limited water infiltration and inhibited O_2_ diffusion (Food and Agriculture Organization of the United Nations [FAO], 2005). The soil in the milpa system had also a higher total N content than the conventional system. Organic rich N was applied in the milpa system while urea was applied in the conventional system, and the latter is more prone to losses, e.g., volatilization, than organic N (e.g., Goyal et al., 1999).

### C and N Mineralization and the Microbial Biomass C

The soil organic matter is the main C substrate for the microbial microorganisms and an increase in soil organic matter will increase emissions of CO_2_ and the amount of N mineralized (e.g., Powlson et al., 1987; Trasar-Cepeda et al., 2008; Krishna, 2014). Kandeler et al. (1999a,b) showed that organic fertilizer applications increased the soil biochemical activity associated with the soil organic matter. This was reflected clearly in the amount of CO_2_ emitted in the aerobic incubation in this study after 45 days. Application of the milpa system had increased the soil organic C content 1.8 times and the CO_2_ emitted from the milpa soil was 2.0 times larger (mean of soil incubated at 5%FC or FC) than emitted from the conventional cultivated soil after 45 days. The amount of N mineralized showed the same trend after 45 days. As such, the amount inorganic N available for the cultivated crops had already increased substantially after 3 years.

The microbial biomass C also showed a small increase (13% in soil incubated at FC) but the field experiment was too short presumably, i.e., only 3 years, to detect larger increases. For instance, Powlson et al. (1987) found increases of 37% and 45% in microbial biomass C when straw was incorporated yearly in two field experiments after 18 years. The ratio biomass-C to soil organic C was 3.7% in the Milpa soil and 5.0% in the conventional tilled soil (mean of soil incubated at 5%FC and FC). These values fall within the range of 0.27% to over 7% reported by Anderson and Domsch (1989). They stated that this wide range was due to differences in soil, vegetation management, sampling time and analytical methods.

Water content is known to affect microbial activity (Gabriel and Kellman, 2014). When a soil becomes too dry, microbial activity ceases and CO_2_ emitted decreases as found in this study. The effect of reduced soil moisture content on the CO_2_ emitted, however, was small in both the milpa and conventional cultivated soil. Incubating the soil at 5%FC reduced the CO_2_ emitted from the milpa soil 1.3 times compared to the soil incubated at FC and 1.1 times in the conventional tilled soil. The semi-arid highlands of Mexico are characterized by an extended dry season so microorganisms are well adapted to prolonged dry conditions.

### Enzymatic Activity

Enzymatic activity in soil is directly related to the microbial biomass, nutrient content, and moisture content (Fließbach et al., 2007). Fuentes-Ponce et al. (2016) reported that the dehydrogenase activity was greater in the milpa soil than in the conventional cultivated soil already after 2 years. In this study, however, the conventional cultivated soil with the lowest microbial biomass C, organic C, total N and moisture content (conventional cultivated 5%FC) showed a larger dehydrogenase activity than the milpa soil. Dehydrogenase activity is related to oxidation of organic matter so oxygen is required (Nannipieri et al., 2003; Wolińska and Stêpniewska, 2011). In this experiment, the soil was incubated in a closed jar and although the flasks were regularly aired anaerobic microsites within the soil could not be excluded especially as the soil was sieved which will break soil aggregates impeding oxygen diffusion. The lower oxygen level in some parts of the soil might have inhibited enzymatic activity (Kandeler et al., 1999b). This might also explain why the hydrogenase activity was higher at 5%FC than at FC.

Phosphatase is recognized as a sensitive indicator of phosphorus stress (Speir and Ross, 1978). Phosphatase activity is greater normally in P deficient soils. In this study phosphatase activity was similar in both agricultural systems. Fuentes-Ponce et al. (2016) reported similar results when determining phosphatase activity in their field study and suggested that this might indicate that the soils were not P deficient.

### Bacterial Community Structure: Effect of Agricultural Practices

Although the two contrasting agricultural systems have been applied for only 3 years, they already had a clear effect on the bacterial community structure, but not on microbial diversity. The two agricultural systems were different in their organic material management and crop rotation. Crop rotation rarely has a significant effect on the bacterial community structure. For instance, Li et al. (2012) reported that a flax-oat-fababean-wheat versus flax-alfalfa-alfalfa-wheat had no significant effect on the bacterial community structure, while Navarro-Noya et al. (2014) reported the same for a maize monoculture versus wheat-maize rotation in the central highlands of Mexico. Changes in the bacterial community due to the crop cultivated can be assumed to be small within 3 years, so the differences between the milpa and conventional cultivated soil in relative abundance of the bacterial groups must have been through the changes in soil organic matter content and composition, i.e., application of organic fertilizer in the milpa system but no such application in the conventional cultivated soil. As a result of organic material application, the soil organic C content had increased and its composition changed, which had altered the bacterial community structure (e.g., Siles et al., 2014).

It has often been reported that organic farming increases microbial diversity (e.g., Hartmann et al., 2015). Lupatini et al. (2016) stated that this is related to the management practices applied, i.e., organic material application, no chemical inputs and biological plant protection. In this study, no such effect on bacterial diversity was found. Similar results have been reported before, e.g., Reilly et al. (2013), but our field experiment might have been too short. i.e., 3 years, to determine a change in the microbial diversity.

Although both systems were dominated by the same bacterial groups, i.e., Acidobacteria, Actinobacteria, Bacteroidetes, and Proteobacteria, the bacterial community structure was different between the milpa and conventional cultivated soil. Acidobacteria, Actinobacteria, Bacteroidetes, and Proteobacteria are often the most abundant in soil (e.g., Li et al., 2012, 2017; Lupatini et al., 2016; Zeng et al., 2016). It is well known that organic farming has an effect on the bacterial community when compared to conventional practices. The relative abundance of only four of the 20 most abundant phyla increased in the milpa soil compared to the conventional cultivated soil at both 5%FC and FC. It can be assumed that Bacteroidetes, Elusimicrobia, FBP and OD1 were favored by the organic fertilizer application in the milpa system and they were not affected by changes in the soil water content. Bacteroidetes have been reported as primary degraders of organic material due to their capacity to mineralize cellulose and copiotrophs (Trivedi et al., 2013; Naas et al., 2014). Elusimicrobia (formerly termite group 1) have been identified as intracellular symbionts of termite gut flagellates but recently (*Endomicrobium proavitum*, class *Endomicrobia*) a free living relative of the intracellular symbionts of termite gut flagellates (Phylum *Elusimicrobia*) was described (Zhenga and Brune, 2015). The gut of termites is an organic material rich environment so the increased abundance in the milpa soil compared to the conventional cultivated soil might be a consequence of the higher organic matter content in the former. Nelson and Stegen (2015) reported after a pan-genome analysis that OD1 have a broad genotypic diversity indicating adaptation to a wide range of growth environments and a high degree of specialization, but the lack of biosynthetic capabilities and DNA repair, along with the presence of potential attachment and adhesion proteins suggest that they are ectosymbionts or parasites of other organisms. Why their relative abundance was higher in the milpa soil than in the conventional cultivated soil was difficult to explain but it might be that they thrived as the higher organic matter content increased growth of microorganisms of which they were parasites.

The relative abundance of six of the 20 most abundant phyla, i.e., AD3, Acidobacteria, Actinobacteria, Cyanobacteria, Fibrobacteres, and Gemmatimonadetes, decreased in the milpa soil compared to the conventional cultivated soil independent of soil water content. The relative abundance of these groups was reduced in the organic rich milpa soil so they thrived in nutrient poor environments and could be considered oligotrophs. Acidobacteria are known to prefer nutrient limited environments. For instance, Thomson et al. (2013) reported that Acidobacteria were favored by bare soil and their relative abundance decreased in rich vegetated soils as found in this study. Actinobacteria, however, have often been described as copiotrophs and are favored when easily decomposable organic material, e.g., dried rice callus (Lee et al., 2011) or wheat residue (Bernard et al., 2007), was applied to soil. Their relative abundance, however, decreased in the milpa soil compared to the conventional cultivated soil. As such, the interaction between soil microorganisms and soil conditions might define how members of a bacterial group respond to a given situation. The effect of the milpa agricultural system on the relative abundance of remaining 10 most abundant bacterial phyla, e.g., Chloroflexi, Firmicutes, Nitrospirae, TM7 and Verrucomicrobia compared to the conventional cultivated soil was controlled by soil water content. Li et al. (2012) reported a higher relative abundance of Proteobacteria under organic farming than in conventional farming, but this depended on the soil water content in this study. The relative abundance of Proteobacteria was higher in the milpa soil than in the conventional cultivated soil when the soil was incubated at FC, but not when incubated at 5%FC. Some of the bacterial phyla that were favored by the milpa system at a certain water content, i.e., 5%FC or FC, but not at the other are known copiotrophs, e.g., Firmicutes (Pascault et al., 2013; Book et al., 2014), or oligotrophs, e.g., Chloroflexi and Verrucomicrobia (Peiffer et al., 2013).

The relative abundance of only two bacterial genera of the 20 most abundant ones (*Aeromicrobium* and *Candidatus* Koribacter) decreased in the milpa soil compared to the conventional cultivated soil independent of soil water content and the relative abundance of only six was always higher in the milpa soil compared to the conventional cultivated soil, i.e., *Agrobacterium*, *Defluvibacter*, *Devosia*, *Ochrobactrum, Ramlibacter*, and *Segetibacter*. The response of the remaining 12, e.g., *Bacillus*, *Flavisolibacter, Pseudomonas*, and *Streptomyces*, to agricultural practices depended on the soil water content. Some of these bacterial groups have been reported to behave as copiotrophs and participate in the degradation of organic material, e.g., *Bacillus* and *Streptomyces* (Garbeva et al., 2003; Cucurachi et al., 2013; Dang-Thuan et al., 2013; Book et al., 2014) or compost formation, e.g., *Flavisolibacter* (Partanen et al., 2010). This confirms once again that bacterial populations are defined by interactions of different factors and a condition that favors a bacterial group, e.g., organic rich milpa soil, alters when a condition changes, i.e., soil water content. This might be the direct result of the changing conditions, i.e., soil water content, on a bacterial group or other bacterial groups are favored replacing other bacterial groups. As such, within the given agroecosystem three different bacterial groups could be distinguished. A first group of bacteria were favored by wet conditions, a second by dry conditions and the response of a third group depended on soil conditions. It remains to be seen if in another agroecosystem, bacterial groups responded in the same way as in the one studied here.

It has to be remembered that data represented are relative abundances of bacterial groups so that the absolute abundance of some bacterial groups remained constant, i.e., there was no positive or negative effect on them, although their relative abundance decreased. In this study, the effect on agricultural practices on the microbial biomass C was small so changes in relative abundances reflected presumably real changes. Although the overall microbial C did not change, fungi are known to better resist drought so they might have replaced bacteria that did not resist drought in the soil incubated at 5%FC (Manzoni et al., 2012). Therefore effects of agricultural practices were reported for soil incubated at both FC and 5%FC.

A possible effect of the bacteria in the manure on the bacterial community structure cannot be discarded (Zhen et al., 2014). The increase in Bacteroidetes might be related to the manure applied, as they are one of the most abundant phyla in cow manure (Shanks et al., 2011) although Li et al. (2017) did not found such an increase when cow manure was applied to soil. Origin and/or composition of the manure might have affected the bacterial community and its possible effect on the soil microbial community (Shanks et al., 2011; Pascault et al., 2013; Zhen et al., 2014).

Li et al. (2012) found a higher relative abundance for *Burkholderia* spp., *Pseudomonas* spp., and *Stenotrophomonas* spp. in organic farming systems compared to conventional practices. In this study, the relative abundance of *Burkholderia* was higher in the milpa (0.22%) than in the conventional tilled soil at FC (0.02%). Suárez-Moreno et al. (2012) in their review of *Burkholderia* described them as diverse, distributed widely and mostly known as pathogens although more than half of the know members of this genus are associated with plants and might be beneficial to them such as in quorum sensing, N_2_ fixation and/or nodulation, and the degradation of aromatic compounds. This last capacity might favor them in an organic rich environment. How members of *Pseudomonas* and *Stenotrophomonas* responded to the agricultural practices depended on the water content of the soil. The relative abundance of *Pseudomonas* was lower in the milpa soil than in the conventional cultivated soil when incubated at FC, but higher when incubated at 5%FC. The relative abundance of *Stenotrophomonas* showed an opposite trend. Other bacterial genera, e.g., *Agrobacterium*, *Flavobacterium*, and *Ramlibacter*, were favored by the organic milpa systems compared to the conventional cultivated soil. Members of *Agrobacterium* are colonizers of root surfaces (Matthysse, 2014) and are cellulose producers (Augimeri et al., 2015).

### Bacterial Community Structure: Effect of Soil Water Content

Soil water content had an effect on the bacterial community structure (e.g., Fuchslueger et al., 2014; Santos-Medellín et al., 2017). Members of the Actinobacteria, Bacteroidetes, Firmicutes, and Verrucomicrobia were favored by dry conditions in the both soils, while those of Acidobacteria, AD3, Chloroflexi and OD1 by wet conditions. Bacteria have different mechanisms to withstand drought. Gram-positive bacteria with their peptidoglycan layer in their cell walls should be more resistant to drought than Gram-negative bacteria (Lennon et al., 2012). Strains that are capable to produce biofilms, i.e., exopolymeric substances such as polysaccharides, nucleic acids, lipids and proteins, and especially biofilms that are hydrophilic helps bacteria to resist better fluctuations in soil water content (Roberson and Firestone, 1992). The production of biofilms, however, comes as an energetic cost, which might reduce growth. Additionally, microfilms hamper gas diffusion so in low O_2_ environments its production might be limited. Microorganisms use also other mechanisms to combat drought stress such as the production of certain components to maintain osmotic equilibrium in their cells, altering their intracellular stoichiometry to reduce oxidative damage or enter a reversible state of dormancy (Lennon et al., 2012). Of course, a combination of the above mentioned factors and soil characteristics will define how bacterial groups respond to drought. Gram-positive bacteria such as Actinobacteria and Firmicutes were more resistant to drought than Gram-negative bacteria such as Acidobacteria in this study, but the Gram-negative Bacteroidetes and Verrucomicrobia also resisted dry conditions. Lennon et al. (2012) found that Firmicutes were also drought resistant but they found that Actinobacteria had a “relative wet optimum.” Santos-Medellín et al. (2017) found that Acidobacteria were depleted in dry conditions and Alphaproteobacteria, Bacilli, and Planctomycetes favored in wet conditions as found in this study, except for the Alphaproteobacteria in the milpa soil. Phylotypes belonging to Actinobacteria and Chloroflexi were enriched in dry conditions and this has been related to their thick cell walls, filamentous growth and spore formation all of which favor them in dry conditions (Santos-Medellín et al., 2017). In this study, Actinobacteria were enriched by dry conditions, but not Chloroflexi so another factor controlled their lack of drought resistance. Some of the most abundant phyla, such as Proteobacteria and five other phyla, were favored by wet conditions in the milpa soil, but by dry conditions in the conventional cultivated soil, while the Gemmatimonadetes and five other phyla showed an opposite trend. Of the 20 most abundant bacterial genera, six were favored by wet conditions independent of soil conditions, e.g., *Pseudomonas, Kaistobacter*, and *Flavisolibacter*, and six were favored by dry conditions, e.g., *Bacillus*, *Steroidobacter*, and *Streptomyces*. Some of the latter are known to be drought resistant and spore forming. Members of *Streptomyces* are drought resistant and their moisture requirements are low, they form arthrospores, and their metabolic capability allow them to grow in composts, plants and water (Hasani et al., 2014). Members of *Bacillus* are aerobic rod shaped bacteria that are able to degrade substrates derived from plant and animal sources like starch, cellulose and hydrocarbons (Slepecky and Hemphill, 2006). They form endospores which forming process is triggered by cellular dehydration so that they can survive dry conditions. *Arthrobacter crystallopoites* can sustain extreme desiccation (Boylen, 1973) and in this study it the relative abundance of *Arthrobacter* was 0.059 for the milpa soil incubated at 5%FC and only 0.003% at FC, and 0.027% in the conventional cultivated soil at 5%FC at 0.005%FC. Of the 20 most important bacterial genera, however, the response of eight of them to soil water content was controlled by agricultural practices, e.g., *Agrobacterium*, *Balneimonas*, *Bradyrhizobium*, *Flavobacterium*, and *Nitrospira*. Surprisingly they were all favored by dry conditions in the conventional tilled soil, but by wet conditions in the milpa soil.

### Bacterial Metabolic Capacity

In soil, nitrification is favored by higher O_2_ content, i.e., an aerobic process, while denitrification requires low O_2_ environments, i.e., an anaerobic process (Wu et al., 2015). Consequently, ammonium oxidation was favored at 5%FC, while denitrification at FC in both soils. Crop residue was left on the soil in the Milpa system, but removed in the conventional tilled soil, and the first was amended additionally with cow dung. The lignin content of crop residue and cow dung is generally low (Frei, 2013), but it was large enough to increase the lignin degrading capacity in the milpa system compared to the conventional tilled soil after only 3 years. The organic material added in the milpa system will provide C substrate for the soil microorganisms so that they will be more active and grow faster than in the conventional system. The soil microbial C content was similar in both systems when the soil was incubated at FC, so the turnover of biomass C was larger in the milpa system than in the conventional system. Consequently, the biomass degrading capacity was larger in the milpa system than in the conventional system.

### Ecological Coherence of High Bacterial Ranks

Philippot et al. (2010) investigated the ecological coherence of high bacterial taxa (from genus to phylum) through genome analysis. They concluded that some bacterial taxa were coherent. Our study did not allow to support this hypothesis when considering the effect of drying on the relative abundance of different taxonomic levels considering a conventional cultivated soil and an organic milpa system. Firmicutes responded most coherently to drying and they were generally favored by drying, although the relative abundance of the Clostridia decreased in the milpa soil, but not in the conventional tilled soil. Santos-Medellín et al. (2017) reported also that the relative abundance of the Clostridia decreased in three soils, while that of the Bacilli increased. They attributed this to the fact that Clostridia are strictly anaerobic so drying increased the concentration of oxygen in soil. However, as evidenced from this study another factor presumably related to the effect of soil management practices must have controlled how this bacterial group responded to drought. Barnard et al. (2013) reported an ecological coherence in the Actinobacteria and Acidobacteria to extreme desiccation and rewetting, which was not found in this study. It has to be remembered that the severity of the drought stress applied to the bacterial communities and soil conditions will also affect how they will respond.

## Conclusion

The organic fertilizer application, crop residue retention and minimum tillage had increased the organic matter content even within a short 3 years compared to the conventional cultivated soil. This increase in soil organic material increased soil microbial activity as evidenced by the increase in C mineralization. The increased organic material decomposition in the milpa system doubled the release of plant nutrients within 56 days compared to the conventional tilled soil. The potential enzymatic activity studied did not reflect the increase in C mineralization, but the experimental incubation conditions might have contributed to this. The agricultural practices and the soil water content controlled the bacterial community structure in soil, but the effect of one factor was often defined by the other factor. The relative abundance of the Gemmatimonadetes was larger in the conventional cultivated soil than in the milpa soil, while that of the Bacteroidetes showed an opposite trend, but that of the Bacteroidetes, FBP, Nitrospirae and Proteobacteria, was also affected by cultivation technique but controlled by soil water content. The relative abundance of the Acidobacteria was lower in the milpa and conventional soil incubated at 5%FC than at FC, but that of Actinobacteria, Cyanobacteria, FBP, Firmicutes, Gemmatimonadetes, and Proteobacteria, was affected by water content, but dependent on cultivation technique applied to the soil. The xenobiotics biodegradation and metabolism functionality was significantly higher in the milpa soil than in the conventional cultivated soil when incubated at FC and carbohydrate metabolism showed an opposite trend. A range of metabolic functions was affected by soil water content, but the effect was different between the milpa and conventional cultivated soil.

It was hypothesized that the higher organic matter content in the milpa soil would protect microorganisms better against drying compared to a conventional cultivated soil. However, the larger organic matter content in the milpa soil did not prevent large changes in the bacterial community structure when the soil was dried to 5%FC. It has to be remembered, however, that under field conditions, the higher organic matter content in the milpa soil will reduce water losses compared to conventional tilled soil. It would be interesting to investigate in the field if the higher water retention in the milpa soil will reduce drought stress on the microbial community compared to the conventional cultivated soil.

## Author Contributions

IM-E designed the experiments, conducted the laboratory experiments, analyzed the data, and wrote the manuscript. MF-G measured the enzyme activity. ML-G conducted the laboratory experiments and wrote the manuscript. DR-V and BR-B conducted the laboratory experiments. ADL-L and SG-A analyzed the data. EG-T extracted DNA and analyzed the data. YN-N and LD analyzed the data and wrote the manuscript. LS-R and JM-J maintained the field experiment. MF-P designed the experiments and wrote the manuscript.

## Conflict of Interest Statement

The authors declare that the research was conducted in the absence of any commercial or financial relationships that could be construed as a potential conflict of interest.

## Footnotes

1 http://www.metagenassist.ca

2 http://picrust.github.io

3 http://rdp.cme.msu.edu/classifier/classifier.jsp
