# Supplementary material for: The Bacterial Community Structure and Microbial Activity in a Traditional Organic Milpa Farming System Under Different Soil Moisture Conditions
Source: Front Microbiol. 2018 Nov 14;9:2737. doi: 10.3389/fmicb.2018.02737 (PMC6246654; doi:10.3389/fmicb.2018.02737)
Supplement: Supplementary file 1 [file Table_1.DOCX]

Table S1. Effect of agricultural practice, i.e. conventional tillage, crop residues removal, chemical fertilizer and herbicide application and monoculture of maize (*Zea mays* L.), or cultivated with the organic milpa system, i.e. zero tillage, retention of crop residues, organic fertilizer application, weed management and crop rotation of maize, pumpkin (*Cucurbita* sp*.*) and beans ([*Phaseolus vulgaris*](https://en.wikipedia.org/wiki/Phaseolus_vulgaris) L.), for three years, water content (WC) of the incubated soil and their interaction (Soil*WC) on the biomass C content, the CO_2_ emission and enzyme activity for soil incubated for 45 days.

| **⎯⎯⎯⎯⎯⎯⎯⎯⎯⎯⎯⎯⎯⎯⎯⎯⎯⎯⎯⎯⎯⎯⎯⎯⎯⎯⎯⎯⎯⎯⎯⎯⎯⎯⎯⎯⎯⎯** | | | | | | | |
| --- | --- | --- | --- | --- | --- | --- | --- |
|  | CO_2_ emitted ^a^ | | Biomass C | Dehydrogenase | Urease | Protease | Acid phosphatase |
| Factor | ⎯⎯⎯⎯⎯⎯⎯⎯⎯⎯⎯⎯⎯⎯ (*p* value) ⎯⎯⎯⎯⎯⎯⎯⎯⎯⎯⎯⎯⎯⎯⎯ | | | | | | |
| ⎯⎯⎯⎯⎯⎯⎯⎯⎯⎯⎯⎯⎯⎯⎯⎯⎯⎯⎯⎯⎯⎯⎯⎯⎯⎯⎯⎯⎯⎯⎯⎯⎯⎯⎯⎯⎯⎯ | | | | | | | |
| Soil | <0.001 | 0.002 | | <0.001 | 0.848 | 0.350 | 0.642 |
| WC | 0.314 | 0.134 | | <0.001 | <0.001 | <0.001 | 0.027 |
| Soil*WC | 0.388 | 0.109 | | 0.011 | 0.509 | 0.981 | 0.066 |
| ⎯⎯⎯⎯⎯⎯⎯⎯⎯⎯⎯⎯⎯⎯⎯⎯⎯⎯⎯⎯⎯⎯⎯⎯⎯⎯⎯⎯⎯⎯⎯⎯⎯⎯⎯⎯⎯⎯ | | | | | | | |
| ^a^ CO_2_ emitted after 45 days. | | | | | | | |
| **⎯⎯⎯⎯⎯⎯⎯⎯⎯⎯⎯⎯⎯⎯⎯⎯⎯⎯⎯⎯⎯⎯⎯⎯⎯⎯⎯⎯⎯⎯⎯⎯⎯⎯⎯⎯⎯⎯** | | | | | | | |
