# Supplementary material for: The Bacterial Community Structure and Microbial Activity in a Traditional Organic Milpa Farming System Under Different Soil Moisture Conditions
Source: Front Microbiol. 2018 Nov 14;9:2737. doi: 10.3389/fmicb.2018.02737 (PMC6246654; doi:10.3389/fmicb.2018.02737)
Supplement: Supplementary file 2 [file Table_2.DOCX]

Table S2. Alpha diversity of the bacterial populations in soil cultivated conventionally, i.e. conventional tillage, crop residues removal, chemical fertilizer and herbicide application and monoculture of maize (*Zea mays* L.), or cultivated with an organic milpa system, i.e. zero tillage, retention of crop residues, organic fertilizer application, weed management and crop rotation of maize, pumpkin (*Cucurbita* sp*.*) and beans ([*Phaseolus vulgaris*](https://en.wikipedia.org/wiki/Phaseolus_vulgaris) L.), for three years. Soil was incubated aerobically for 45 days.

| ⎯⎯⎯⎯⎯⎯⎯⎯⎯⎯⎯⎯⎯⎯⎯⎯⎯⎯⎯⎯⎯⎯⎯⎯⎯⎯⎯⎯⎯⎯⎯⎯⎯⎯⎯⎯⎯⎯⎯⎯⎯⎯⎯⎯⎯⎯⎯⎯⎯⎯⎯⎯⎯⎯⎯⎯ | | | | | | | | | | |
| --- | --- | --- | --- | --- | --- | --- | --- | --- | --- | --- |
|  | Water | Incubation | | Total | Goods’ | | Observed | Chao1 richness | Simpson | Shannon |
| Treatment | content | (days) | | sequences ^a^ | coverage (%) | | OTUs ^bc^ | estimator ^c^ | diversity index ^c^ | diversity index ^c^ |
| ⎯⎯⎯⎯⎯⎯⎯⎯⎯⎯⎯⎯⎯⎯⎯⎯⎯⎯⎯⎯⎯⎯⎯⎯⎯⎯⎯⎯⎯⎯⎯⎯⎯⎯⎯⎯⎯⎯⎯⎯⎯⎯⎯⎯⎯⎯⎯⎯⎯⎯⎯⎯⎯⎯⎯⎯ | | | | | | | | | | |
| Conventional | 5%FC ^d^ | | 0 | 8165 | 86 | 730 | | 1818 | 0.998 | 9.20 |
|  |  | 1 | | 6238 | 85 | 652 | | 1492 | 0.996 | 8.90 |
|  |  | 3 | | 1362 | 72 | 540 | | 1183 | 0.996 | 8.54 |
|  |  | 7 | | 4681 | 92 | 420 | | 661 | 0.995 | 8.15 |
|  |  | 14 | | 3845 | 93 | 373 | | 642 | 0.994 | 7.90 |
|  |  | 45 | | 1427 | 89 | 283 | | 481 | 0.983 | 7.07 |
|  | FC | 0 | | 8144 | 91 | 492 | | 1218 | 0.984 | 7.76 |
|  |  | 1 | | 4755 | 86 | 591 | | 1201 | 0.995 | 8.65 |
|  |  | 3 | | 2872 | 83 | 543 | | 1074 | 0.996 | 8.57 |
|  |  | 7 | | 2656 | 93 | 317 | | 527 | 0.991 | 7.55 |
|  |  | 14 | | 2194 | 92 | 293 | | 552 | 0.990 | 7.40 |
|  |  | 45 | | 1741 | 92 | 272 | | 439 | 0.981 | 7.06 |
| ⎯⎯⎯⎯⎯⎯⎯⎯⎯⎯⎯⎯⎯⎯⎯⎯⎯⎯⎯⎯⎯⎯⎯⎯⎯⎯⎯⎯⎯⎯⎯⎯⎯⎯⎯⎯⎯⎯⎯⎯⎯⎯⎯⎯⎯⎯⎯⎯⎯⎯⎯⎯⎯⎯⎯⎯ | | | | | | | | | | |

| ⎯⎯⎯⎯⎯⎯⎯⎯⎯⎯⎯⎯⎯⎯⎯⎯⎯⎯⎯⎯⎯⎯⎯⎯⎯⎯⎯⎯⎯⎯⎯⎯⎯⎯⎯⎯⎯⎯⎯⎯⎯⎯⎯⎯⎯⎯⎯⎯⎯⎯⎯⎯⎯⎯⎯⎯ | | | | | | | | |
| --- | --- | --- | --- | --- | --- | --- | --- | --- |
| Milpa | 5%FC | 0 | 3803 | 81 | 643 | 1356 | 0.997 | 8.96 |
|  |  | 1 | 3829 | 84 | 546 | 1114 | 0.985 | 8.19 |
|  |  | 3 | 1135 | 73 | 495 | 1113 | 0.995 | 8.31 |
|  |  | 7 | 5300 | 94 | 411 | 713 | 0.995 | 8.14 |
|  |  | 14 | 3356 | 91 | 410 | 671 | 0.994 | 8.05 |
|  |  | 45 | 3233 | 92 | 355 | 622 | 0.994 | 7.82 |
|  | FC | 0 | 7841 | 88 | 636 | 1417 | 0.996 | 8.85 |
|  |  | 1 | 7211 | 87 | 595 | 1284 | 0.985 | 8.35 |
|  |  | 3 | 1638 | 81 | 479 | 919 | 0.995 | 8.35 |
|  |  | 7 | 5759 | 94 | 435 | 755 | 0.994 | 8.14 |
|  |  | 14 | 2357 | 91 | 334 | 611 | 0.991 | 7.58 |
|  |  | 45 | 2943 | 91 | 357 | 657 | 0.993 | 7.79 |
| ⎯⎯⎯⎯⎯⎯⎯⎯⎯⎯⎯⎯⎯⎯⎯⎯⎯⎯⎯⎯⎯⎯⎯⎯⎯⎯⎯⎯⎯⎯⎯⎯⎯⎯⎯⎯⎯⎯⎯⎯⎯⎯⎯⎯⎯⎯⎯⎯⎯⎯⎯⎯⎯⎯⎯⎯ | | | | | | | | |
| ^a^ Combined reads of three replicates. High quality reads > 25 Q-score, containing no barcode errors and homopolymers, ^b^ OTU-97% Operational taxonomic unit as determined at a similarity threshold of 97%, ^c^ Calculated with rarified data sets to 1,135 reads, ^d^ FC: Field capacity. | | | | | | | | |
| ⎯⎯⎯⎯⎯⎯⎯⎯⎯⎯⎯⎯⎯⎯⎯⎯⎯⎯⎯⎯⎯⎯⎯⎯⎯⎯⎯⎯⎯⎯⎯⎯⎯⎯⎯⎯⎯⎯⎯⎯⎯⎯⎯⎯⎯⎯⎯⎯⎯⎯⎯⎯⎯⎯⎯⎯ | | | | | | | | |
