# Supplementary material for: The Bacterial Community Structure and Microbial Activity in a Traditional Organic Milpa Farming System Under Different Soil Moisture Conditions
Source: Front Microbiol. 2018 Nov 14;9:2737. doi: 10.3389/fmicb.2018.02737 (PMC6246654; doi:10.3389/fmicb.2018.02737)
Supplement: Supplementary file 3 [file Table_3.DOC]

Table S3. Effect of agricultural practices, soil water content and their interaction on the relative abundance of the 20 most abundant bacterial phyla (mean relative abundance phyla ≥ 0.03) and genus (relative abundance ≥ 0.11).

|  | | | | | | | | | | | | |  |
| --- | --- | --- | --- | --- | --- | --- | --- | --- | --- | --- | --- | --- | --- |
|  | Conventional | | Milpa | | 5%FC vs FC | | Conv vs Milpa | | Soil | WC | Interaction | |  |
|  |  | |  | |  | |  | |  |  |  | |  |
|  | 5%FC | FC | 5%FC | FC | Sco | Smo | 5%FC | FC | smc vs smo | 5%FC vs FC | Soil vs WC | |  |
| Phylum |  Relative abundance (%)  | | | |  *p* value  | | | | | | | |  |
|  | | | | | | | | | | | | |  |
| Acidobacteria | 22.54 | 27.38 | 19.70 | 25.42 | **0.044** | **0.012** | 0.118 | 0.355 | 0.109 | **0.006** | 0.937 | |  |
| Actinobacteria | 11.96 | 11.30 | 11.08 | 7.45 | 0.406 | **0.044** | 0.462 | 0.085 | 0.104 | 0.067 | 0.403 | |  |
| AD3 | 0.00 | 0.18 | 0.00 | 0.02 | 0.345 | 0.345 | NA | 1.000 | ND | ND | ND | |  |
| Armatimonadetes | 0.92 | 1.42 | 1.42 | 0.92 | 0.580 | 0.200 | 0.235 | 0.496 | 0.758 | 0.665 | 0.262 | |  |
| Bacteroidetes | 11.27 | 10.07 | 16.81 | 13.82 | 0.279 | 0.265 | **0.006** | **0.019** | **0.001** | 0.166 | 0.879 | |  |
| BRC1 | 0.07 | 0.14 | 0.22 | 0.10 | 0.801 | 0.746 | 0.356 | 0.755 | 0.227 | 0.595 | 0.542 | |  |
| Chlorobi | 0.33 | 0.90 | 0.48 | 0.37 | 0.144 | 0.470 | 0.424 | 0.161 | 0.308 | 0.447 | 0.247 | |  |
| Chloroflexi | 5.05 | 7.99 | 5.58 | 7.34 | **0.020** | 0.192 | 0.767 | 0.650 | 0.892 | **0.014** | 0.507 | |  |
| Cyanobacteria | 1.80 | 0.91 | 1.70 | 0.66 | 0.189 | **<0.001** | 0.477 | 0.837 | 0.884 | **0.017** | 0.696 | |  |
| Elusimicrobia | 0.14 | 0.06 | 0.43 | 1.04 | 0.541 | 0.958 | 0.560 | 0.349 | 0.303 | 0.899 | 0.609 | |  |
| FBP | 0.34 | 0.13 | 0.64 | 0.65 | **0.024** | 0.962 | 0.099 | **<0.001** | **0.003** | 0.389 | 0.691 | |  |
| Fibrobacteres | 0.06 | 0.03 | 0.01 | 0.03 | 0.180 | 0.931 | 0.394 | 0.621 | 0.598 | 0.598 | 0.598 | |  |
| Firmicutes | 4.57 | 3.49 | 5.03 | 1.65 | 0.079 | **<0.001** | 0.461 | 0.104 | 0.695 | **0.001** | 0.151 | |  |
| Gemmatimonadetes | 5.12 | 5.51 | 3.17 | 2.09 | 1.000 | **0.019** | **0.002** | **<0.001** | **0.001** | 0.233 | 0.285 | |  |
| Nitrospirae | 1.31 | 1.08 | 0.70 | 1.73 | 0.402 | 0.241 | **0.018** | 0.669 | **0.027** | 0.929 | 0.168 | |  |
| OD1 | 0.06 | 0.16 | 0.17 | 0.23 | 0.697 | 0.936 | 0.361 | 0.574 | 0.236 | 0.865 | 0.957 | |  |
| Planctomycetes | 1.26 | 0.93 | 1.37 | 0.82 | 0.198 | 0.064 | 0.457 | 0.645 | 0.561 | **0.016** | 0.779 | |  |
| Proteobacteria | 25.48 | 21.28 | 22.70 | 28.21 | **0.040** | 0.134 | 0.389 | **0.009** | 0.082 | 0.696 | 0.015 | |  |
| TM7 | 1.19 | 1.23 | 1.42 | 1.19 | 0.776 | 0.223 | 0.154 | 0.669 | 0.158 | 0.293 | 0.347 | |  |
| Verrucomicrobia | 1.30 | 0.54 | 0.86 | 0.55 | 0.162 | 0.069 | 0.862 | 0.739 | 0.708 | 0.066 | 0.648 | |  |
|  | | | | | | | | | | | |  | |

Table S2. Continued.

|  | | | | | | | | | | | | |
| --- | --- | --- | --- | --- | --- | --- | --- | --- | --- | --- | --- | --- |
| Genus |  |  |  |  |  |  |  |  |  |  |  | |
|  | | | | | | | | | | | | |
| *Aeromicrobium* | 0.77 | 0.81 | 0.36 | 0.73 | 0.936 | 0.616 | 0.527 | 0.688 | 0.369 | 0.826 | 0.979 | |
| *Agrobacterium* | 0.09 | 0.05 | 0.46 | 0.81 | 0.659 | 0.250 | 0.068 | **<0.001** | **0.015** | 0.533 | 0.464 | |
| *Bacillus* | 3.07 | 2.04 | 4.41 | 1.19 | 0.074 | **<0.001** | 0.098 | 0.195 | 0.491 | **0.001** | 0.069 | |
| *Balneimonas* | 0.36 | 0.26 | 0.26 | 0.83 | 0.424 | 0.141 | 0.987 | 0.050 | 0.198 | 0.486 | 0.119 | |
| *Bradyrhizobium* | 0.70 | 0.34 | 0.22 | 0.47 | **0.042** | 0.065 | **0.013** | 0.163 | 0.236 | 0.390 | **0.014** | |
| *Candidatus* Koribacter | 0.68 | 0.41 | 0.23 | 0.14 | 0.623 | 0.370 | **0.024** | **0.033** | **0.021** | 0.721 | 0.828 | |
| *Cryseobacterium* | 0.15 | 0.93 | 0.17 | 0.23 | 0.386 | **0.035** | 0.225 | 0.986 | 0.908 | 0.204 | 0.879 | |
| *Defluvibacter* | 0.46 | 0.42 | 0.74 | 0.84 | 0.782 | 0.552 | 0.380 | 0.259 | 0.103 | 0.876 | 0.541 | |
| *Devosia* | 0.21 | 0.09 | 0.63 | 0.41 | 0.296 | 0.924 | 0.224 | 0.057 | 0.100 | 0.981 | 0.434 | |
| *Flavisolibacter* | 2.46 | 2.53 | 2.43 | 3.89 | 0.874 | **0.046** | 0.934 | 0.060 | 0.164 | 0.252 | 0.126 | |
| *Flavobacterium* | 0.41 | 0.05 | 0.34 | 0.54 | 0.100 | 0.761 | 0.404 | **0.019** | 0.083 | 0.565 | 0.304 | |
| *Kaistobacter* | 0.25 | 0.50 | 0.12 | 0.76 | 1.000 | **0.009** | 0.286 | 0.112 | 0.520 | 0.302 | 0.141 | |
| *Nitrospira* | 0.57 | 0.56 | 0.28 | 0.69 | 0.726 | 0.261 | 0.366 | 0.646 | 0.241 | 0.407 | 0.772 | |
| *Ochrobactrum* | 1.63 | 1.80 | 2.06 | 2.12 | 0.124 | 0.880 | 0.100 | 0.826 | 0.440 | 0.988 | 0.515 | |
| *Pseudomonas* | 0.35 | 0.94 | 0.47 | 0.60 | 0.987 | 0.974 | 0.967 | 0.883 | 0.992 | 0.874 | 0.767 | |
| *Ramlibacter* | 0.37 | 0.22 | 0.68 | 1.18 | 0.127 | 0.125 | 0.187 | **<0.001** | **0.002** | 0.574 | 0.078 | |
| *Segetibacter* | 1.15 | 0.65 | 1.90 | 1.29 | **0.026** | 0.203 | 0.235 | 0.071 | **0.037** | **0.021** | 0.813 | |
| *Stenotrophomonas* | 0.86 | 0.85 | 0.54 | 0.98 | 0.902 | 0.329 | 0.956 | 0.296 | 0.182 | 0.457 | 0.642 | |
| *Steroidobacter* | 0.67 | 0.18 | 0.64 | 0.45 | 0.307 | 0.441 | 0.337 | 0.187 | **0.049** | 0.280 | 0.799 | |
| *Streptomyces* | 0.46 | 0.21 | 0.92 | 0.10 | **0.042** | **0.002** | 1.000 | 0.223 | 0.998 | **0.014** | 0.371 | |
|  | | | | | | | | | | | |  |
| ^a^ 5%FC: soil incubated at 5% field capacity, ^b^ FC: soil incubated at field capacity, ^c^ sco: conventional agriculture , ^d^ smo: milpa system. | | | | | | | | | | | |  |
|  | | | | | | | | | | | |  |
