# Supplementary material for: The Bacterial Community Structure and Microbial Activity in a Traditional Organic Milpa Farming System Under Different Soil Moisture Conditions
Source: Front Microbiol. 2018 Nov 14;9:2737. doi: 10.3389/fmicb.2018.02737 (PMC6246654; doi:10.3389/fmicb.2018.02737)
Supplement: Supplementary file 4 [file Table_4.DOC]

Table S4. Analysis of similarities (ANOSIM) based on weighted UniFrac pairwise distances, testing for differences in the bacterial communities between soil cultivated conventionally, i.e. conventional tillage, crop residues removal, chemical fertilizer and herbicide application and monoculture of maize (*Zea mays* L.), or an organic milpa system, i.e. zero tillage, retention of crop residues, organic fertilizer application, weed management and crop rotation of maize, pumpkin (*Cucurbita* sp*.*) and beans ([*Phaseolus vulgaris*](https://en.wikipedia.org/wiki/Phaseolus_vulgaris) L.), for three years, water content, i.e. at 5% field capacity or at field capacity, and incubation time.

|  | | | |
| --- | --- | --- | --- |
| Comparison | | R | *p* value |
|  | | | |
| Conventional cultivated versus milpa soil | 0.0945 | | < 0.001 |
| Soil incubated at 5%FC versus soil incubated at FC | 0.1399 | | < 0.001 |
| Conventional at 5%FC, conventional at FC, milpa at 5%FC versus milpa at FC | 0.1700 | | < 0.001 |
|  | | | |
| Milpa soil |  | |  |
| Effect of soil moisture content | 0.1932 | | < 0.001 |
| Effect of incubation time | 0.1981 | | < 0.001 |
|  | | | |
| Conventional cultivated soil |  | |  |
| Effect of soil moisture content (soil incubated at 5%FC versus FC) | 0.1312 | | 0.004 |
| Effect of incubation time | 0.0873 | | 0.017 |
|  | | | |
| Soil incubated at 5% field capacity |  | |  |
| Effect of cultivation technique (conventional versus milpa) | 0.1027 | | 0.006 |
| Effect of incubation time | 0.1495 | | 0.002 |
|  | | | |
| Soil incubated at field capacity |  | |  |
| Effect of cultivation technique (conventional versus milpa) | 0.1310 | | 0.002 |
| Effect of incubation time | 0.1191 | | 0.004 |
|  | | | |
| ^a^ FC: Field capacity. | | | |
|  | | | |
