# Supplementary figures and images for: The Bacterial Community Structure and Microbial Activity in a Traditional Organic Milpa Farming System Under Different Soil Moisture Conditions
Source: Front Microbiol. 2018 Nov 14;9:2737. doi: 10.3389/fmicb.2018.02737 (PMC6246654; doi:10.3389/fmicb.2018.02737)

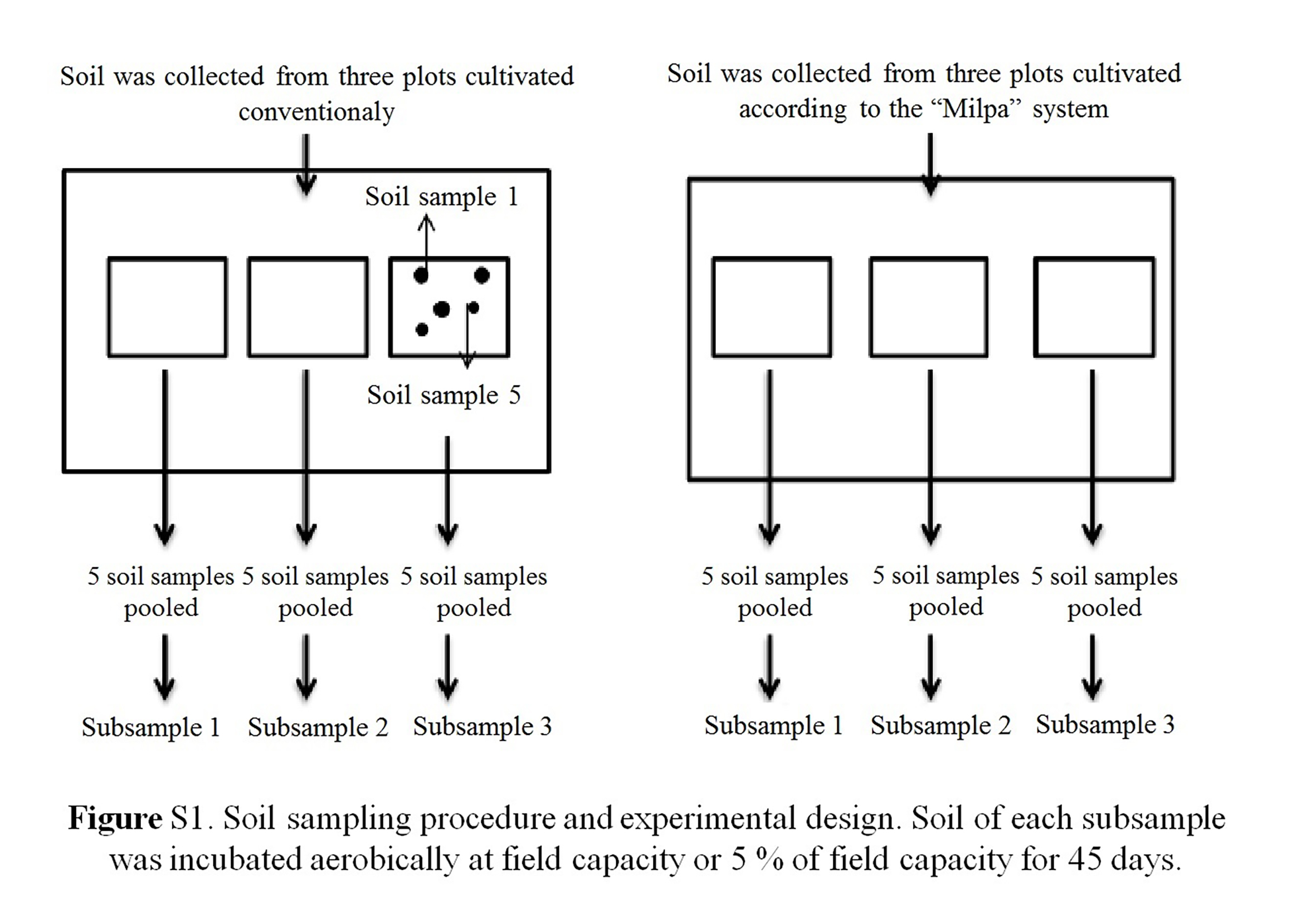

Supplement: Supplementary file 5 [file Image_1.JPEG]

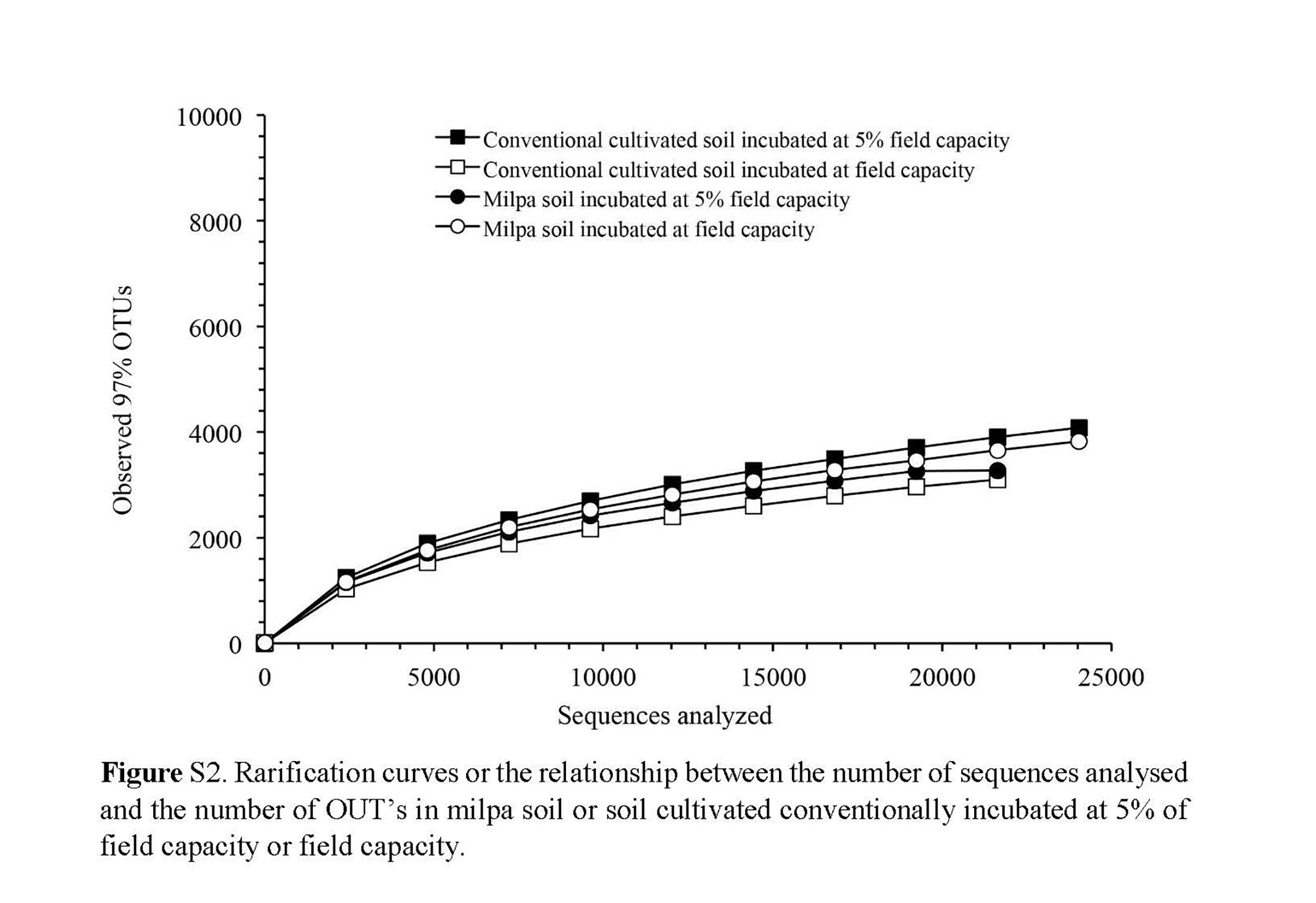

Supplement: Supplementary file 6 [file Image_2.JPEG]

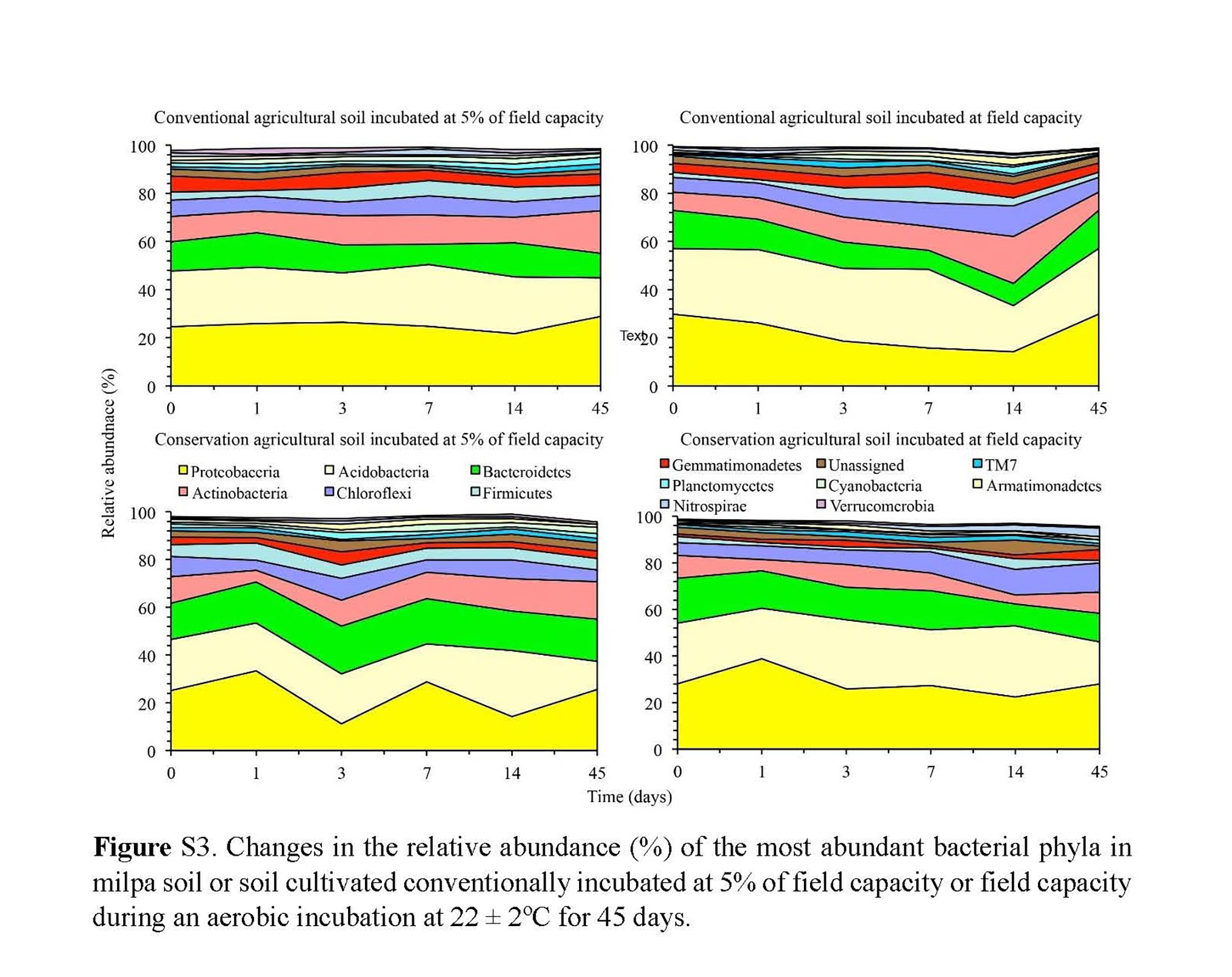

Supplement: Supplementary file 7 [file Image_3.JPEG]
